# Prognostic effect of the preoperative albumin-to-globulin ratio bladder cancer: a meta-analysis

**DOI:** 10.3389/fmed.2026.1848522

**Published:** 2026-08-25

**Authors:** Fangfang Huang, Changyun Chen, Yan Mao, Tingting Lin, Qianqian Hu

**Affiliations:** 1 Department of Urology, The First Affiliated Hospital of Wenzhou Medical University, Wenzhou, China; 2 Department of Orthopedics, The First Affiliated Hospital of Wenzhou Medical University, Wenzhou, China

**Keywords:** AGR, albumin-to-globulin ratio, bladder cancer, meta-analysis, prognosis

## Abstract

**Background:**

The preoperative albumin-to-globulin ratio (AGR) has emerged as a potential prognostic indicator in various malignancies. However, its specific prognostic value in bladder cancer remains to be fully elucidated. Thus, the association between preoperative the AGR and the prognosis of bladder cancer should be systematically evaluated through a meta-analysis of the available evidence.

**Methods:**

A systematic search was performed across major electronic databases (PubMed, Embase, and the Cochrane Library) to identify studies reporting the prognostic significance of AGR in bladder cancer. The primary outcomes were overall survival (OS) and progression-free survival (PFS). Hazard ratios (HRs) and 95% confidence intervals (CIs) were pooled to assess the strength of the association.

**Results:**

A total of 8 studies involving 8305 patients were included in this meta-analysis. Our pooled results found that a low preoperative AGR was significantly associated with poor CSS (HR = 1.25, 95% CI: 1.11–1.38), PFS (HR = 1.92, 95% CI: 1.35–2.50), RFS (HR = 1.36, 95% CI: 1.09–1.64) compared with a high AGR in patients with bladder cancer.

**Conclusion:**

The preoperative AGR is a reliable prognostic factor for bladder cancer, suggesting that it could be integrated into clinical practice for risk assessment.

## Introduction

Bladder cancer is among the most common malignancies of the urinary system worldwide and remains associated with high recurrence and mortality rates despite advances in surgical and perioperative management (1). Accurate prognostic evaluation is essential for optimizing treatment strategies and improving clinical outcomes in patients undergoing surgical treatment for bladder cancer (2, 3).

Growing evidence suggests that systemic inflammation and nutritional status play critical roles in tumor progression and survival (4, 5). Cancer-related inflammation promotes tumor cell proliferation, angiogenesis, invasion, and immune escape, whereas malnutrition is associated with impaired host immunity and poor tolerance to treatment (6, 7). Therefore, inflammation- and nutrition-based biomarkers have attracted increasing attention as prognostic indicators in oncology (8).

The albumin-globulin ratio (AGR), which is calculated from serum albumin and globulin levels, reflects both nutritional condition and systemic inflammatory status (9). Serum albumin is an indicator of nutritional reserve, while elevated globulin levels are associated with chronic inflammatory responses involved in tumor development (10). A decreased AGR has been reported to be correlated with unfavorable survival outcomes in several malignancies, including urological cancers (11).

Several retrospective studies have investigated the prognostic significance of preoperative AGR in bladder cancer (12–14); however, the findings of these studies remain inconsistent because of differences in study design, sample size, and cutoff values. Consequently, the clinical value of the AGR as a prognostic biomarker in bladder cancer has not been fully established. Therefore, this meta-analysis was conducted to systematically evaluate the association between preoperative AGR and prognosis for bladder cancer, aiming to provide more robust evidence regarding its prognostic significance.

## Methods

The study design, literature screening, data extraction, and statistical analyses were performed in accordance with the Preferred Reporting Items for Systematic Reviews and Meta-Analyses (PRISMA 2020) guidelines (15).

### Literature search strategy

A comprehensive literature search was systematically performed in the PubMed, Web of Science, Embase, and Cochrane Library databases from database inception to January, 5, 2026. The search strategy combined Medical Subject Headings (MeSH) terms and free-text keywords related to bladder cancer and albumin-globulin ratio. The search terms used were: “bladder cancer,” “bladder carcinoma,” “urothelial carcinoma,” “albumin globulin ratio,” and “AGR.” Additionally, reference lists of relevant articles and reviews were manually screened to identify potentially eligible studies.

### Inclusion and exclusion criteria

Studies were included if they met the following criteria: (1) patients were pathologically diagnosed with bladder cancer; (2) preoperative AGR was evaluated before treatment; (3) associations between AGR and survival outcomes were reported, including overall survival (OS), cancer-specific survival (CSS), progression-free survival (PFS), or disease-free survival (DFS); (4) hazard ratios (HRs) with 95% confidence intervals (CIs), or sufficient data for their calculation, were available; and (5) cohort or case-control studies. The exclusion criteria were as follows: (1) reviews, case reports, conference abstracts, editorials, or letters; (2) studies lacking survival outcome data; and (3) duplicate publications or overlapping patient cohorts.

### Data extraction and quality assessment

Two investigators independently extracted data from the eligible studies. Any discrepancies were resolved through discussion or consultation with a third reviewer. The following information was collected: first author and publication year, country and study design, sample size, patient characteristics, treatment modality, AGR cutoff value, follow-up duration, survival endpoints (OS, CSS, DFS, or PFS), and Hazard ratios (HRs) and corresponding 95% CIs. The methodological quality of the included studies was evaluated independently by two reviewers using the Newcastle–Ottawa Scale (NOS) (16). Studies with NOS scores ≥ 6 were considered high quality. When the reference category reported in the original study was inconsistent with the predefined comparison direction of this meta-analysis, the effect estimates were recalculated using the method proposed by Hamling et al. This approach allows relative effect estimates and corresponding precision estimates to be derived for alternative comparisons from category-specific estimates sharing a common reference group (17).

### Statistical analysis

All statistical analyses were performed using Stata software (version 12.0; StataCorp, College Station, TX, USA). Hazard ratios (HRs) with 95% confidence intervals (CIs) were pooled to evaluate the association between the preoperative AGR and survival outcomes. Statistical heterogeneity among studies was assessed using Cochran’s Q test and the I^2^ statistic. Significant heterogeneity was defined as I^2^ > 50% or *P* < 0.10. A random-effects model (DerSimonian-Laird method) was applied when significant heterogeneity existed; otherwise, a fixed-effects model was used. Subgroup analyses were conducted according to factors such as sample size, AGR cutoff value, geographic region, and survival endpoint when sufficient data were available. Sensitivity analysis was performed by sequentially omitting individual studies to evaluate the stability of the pooled results. A *P*-value < 0.05 was considered to indicate statistical significance.

## Results

### Study selection

**FIGURE 1:**
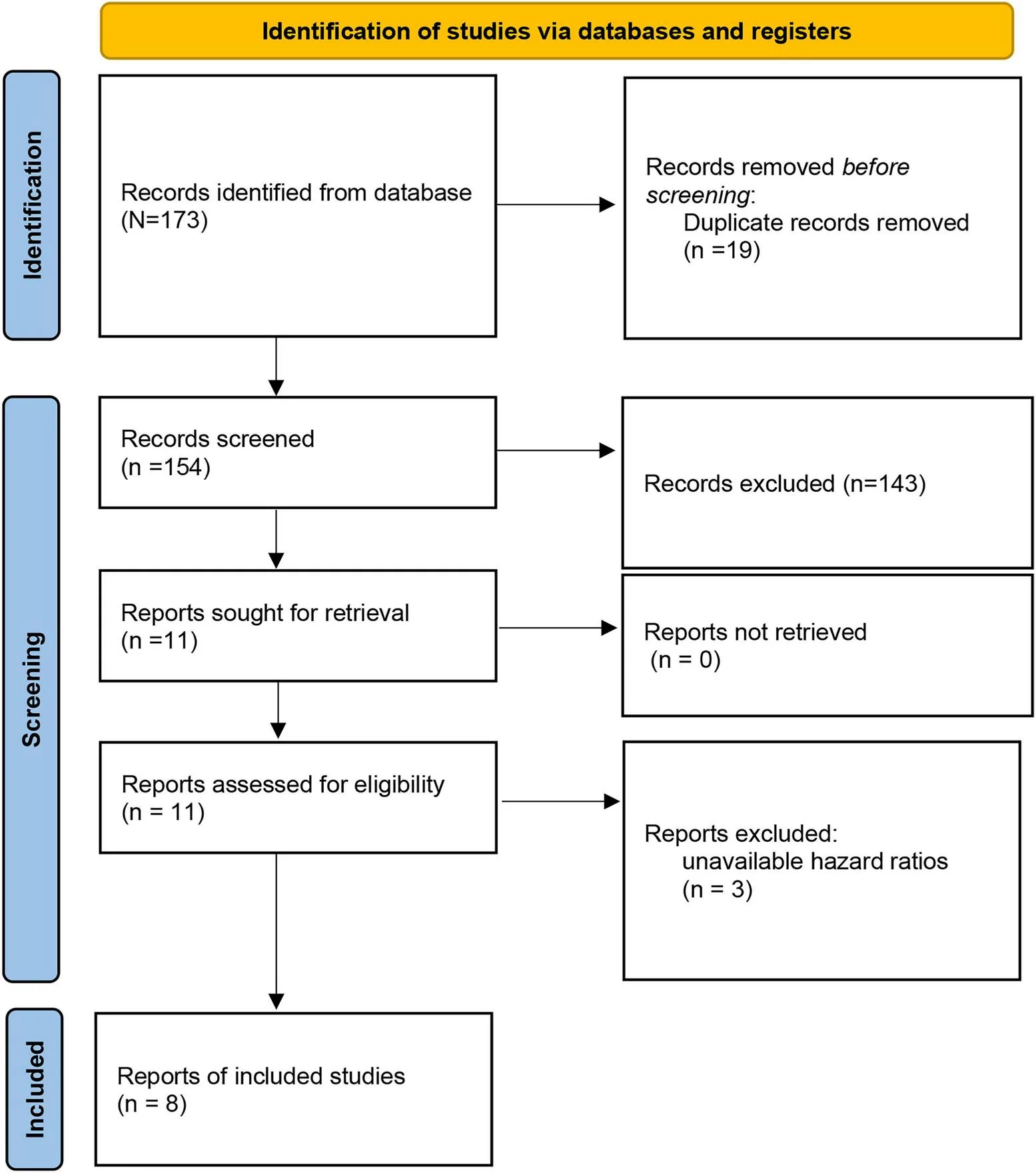
Flow diagram of literature search.

A total of 173 studies were initially identified through database searching, including 44 records from PubMed, 127 from Embase, and 2 from the Cochrane Library. After 19 duplicate records were removed, 154 studies remained for title and abstract screening. Subsequently, 143 articles were excluded because of irrelevance, review article status, case-report design, or insufficient data. After the full-text assessment of 11 potentially eligible studies, 3 studies were further excluded because hazard ratios were unavailable. Finally, 8 studies (12–14, 18–22) involving 8305 patients were included in the quantitative meta-analysis. The detailed study selection process is illustrated in the PRISMA flow diagram (Figure 1). The main characteristics of the included studies are summarized in Table 1. All eligible studies were retrospective cohort studies published between 2017 and 2026. The sample sizes ranged from 176 to 4335 patients. The studies included were conducted in Austria, Japan, China, and Korea, with most patients undergoing radical cystectomy or surgical treatment for bladder cancer. The cutoff values of the preoperative AGR varied among the studies and ranged from 1.1 to 1.6. The survival outcomes evaluated included overall survival (OS), cancer-specific survival (CSS), progression-free survival (PFS), disease-free survival (DFS), recurrence-free survival (RFS), and metastasis-free survival (MFS). Hazard ratios were primarily obtained from multivariate analyses in most studies. In accordance with the Newcastle–Ottawa Scale (NOS), all included studies were considered high quality, with scores ranging from 6 to 8.

**TABLE 1:** Characteristics of studies included in the meta-analysis.

References	Country	Study type	Age	Sample size	Tumor type	Treatment modality	Cut off value	Follow-up (months)	Survival outcomes	NOS score
Quhal et al. (19)	Austria	Retrospective	67	1096	NMIBC	TURB	1.41	63.7	PFS, RFS	8
Takada et al. (22)	Japan	Retrospective	71	235	MIBC	RC	1.10	60	OS, DFS	7
Zhang et al. (21)	China	Retrospective	66	127	MIBC	RC	1.55	–	OS, PFS	6
Niwa et al. (13)	Japan	Retrospective	71	364	NMIBC	TURB	1.60	47	RFS	7
Liu et al. (12)	China	Retrospective	–	1676	MIBC	RC	1.55	38	OS, PFS, CSS	7
Oh et al. (14)	Korea	Retrospective	68.05	176	MIBC	RC	1.32	32.4	CSS, MFS	7
Schuettfort et al. (20)	Austria	Retrospective	67.02	4335	–	RC	1.52	31.5	OS, RFS, CSS	7
Liu et al. (18)	China	Retrospective	61.7	296	MIBC	RC	1.60	72	RFS, CSS	6

PFS, progression-free survival; RFS, recurrence-free survival; DFS, disease-free survival; OS, overall survival; CSS, cancer-specific survival; MFS, metastasis-free survival; NOS, Newcastle–Ottawa Scale; TURB, transurethral resection of bladder tumor; RC, radical cystectomy; MIBC, muscle invasive bladder cancer; NIMBC, non-muscle invasive bladder cancer.

### Association between preoperative the AGR and overall survival

**FIGURE 2:**
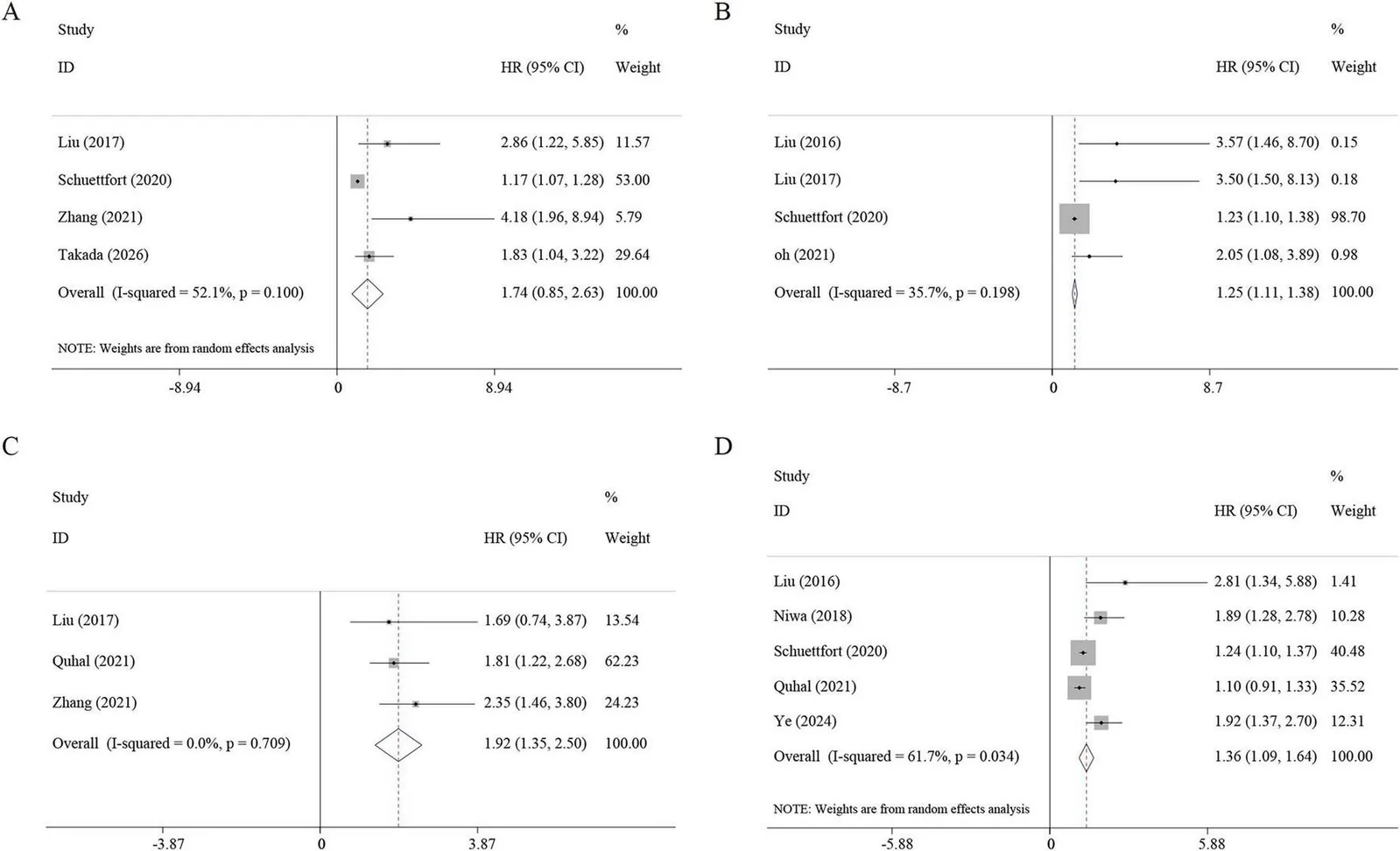
Meta-analysis of the association between AGR and prognosis. **(A)** OS, **(B)** CSS, **(C)** PFS, **(D)** RFS. OS, overall survival; AGR, albumin-to-globulin ratio; CSS, cancer-specific survival; PFS, progression-free survival; RFS, recurrence-free survival. An HR greater than 1 indicates a higher risk of poor prognosis in patients with low AGR.

A total of 4 studies reported data on overall survival (OS). Pooled analysis showed that a low preoperative AGR was not associated with poor overall survival in patients with bladder cancer (HR = 1.74, 95% CI: 0.85–2.63, *P* = 0.055) (Figure 2). Significant heterogeneity was observed among the studies (I^2^ = 52.1%, *P* = 0.10); therefore, a random-effects model was applied.

### Association between the AGR and cancer-specific survival

Four studies evaluated the relationship between AGR and cancer-specific survival (CSS). The pooled results indicated that a decreased preoperative AGR was significantly associated with worse CSS (HR = 1.25, 95% CI: 1.11–1.38, *P* < 0.001) (Figure 2), with low heterogeneity (I^2^ = 35.7%, *p* = 0.198).

### Association between the AGR and progression-free or recurrence-free survival

A pooled analysis of 3 studies assessing progression-related outcomes (PFS), revealed that a lower AGR was correlated with poorer PFS (HR = 1.92, 95% CI: 1.35–2.50, *P* < 0.001) (Figure 2). In addition, the meta-analysis revealed worse RFS for patients with a decreased preoperative AGR with an HR of 1.36 (95% CI 1.09–1.64, *P* < 0.001) (Figure 2).

### Subgroup analysis

Subgroup analysis based on treatment modality revealed that a low AGR was generally associated with poor survival outcomes across different treatment settings (Table 2). Among patients who underwent radical cystectomy, a low AGR was associated with poor CSS, RFS, and PFS. In the TURB-treated cohort, a low AGR was also associated with unfavorable RFS and PFS, although the RFS subgroup showed substantial heterogeneity.

**TABLE 2:** Subgroup analysis based on treatment modality.

Outcomes	Subgroup	No. studies	HR (95% CI)	*P*-value	*I*^2^ (%)	*P*-value
OS	RC	3	1.74 (0.85, 2.63)	0.055	52.1	0.100
CSS	RC	10	1.25 (1.11, 1.38)	0.001	35.7	0.198
RFS	TURB	2	1.41 (0.65, 2.17)	0.264	74.7	0.047
	RC	3	1.60 (0.94, 2.27)	0.037	64.5	0.060
PFS	TURB	1	1.81 (1.08, 2.54)	0.001	–	–
	RC	2	2.11 (1.18, 3.05)	0.001	0	0.508

OR, odds ratio; CI, confidence interval; PFS, progression-free survival; RFS, recurrence-free survival; OS, overall survival; CSS, cancer-specific survival; TURB, transurethral resection of bladder tumor; RC, radical cystectomy.

### Sensitivity analysis

**FIGURE 3:**
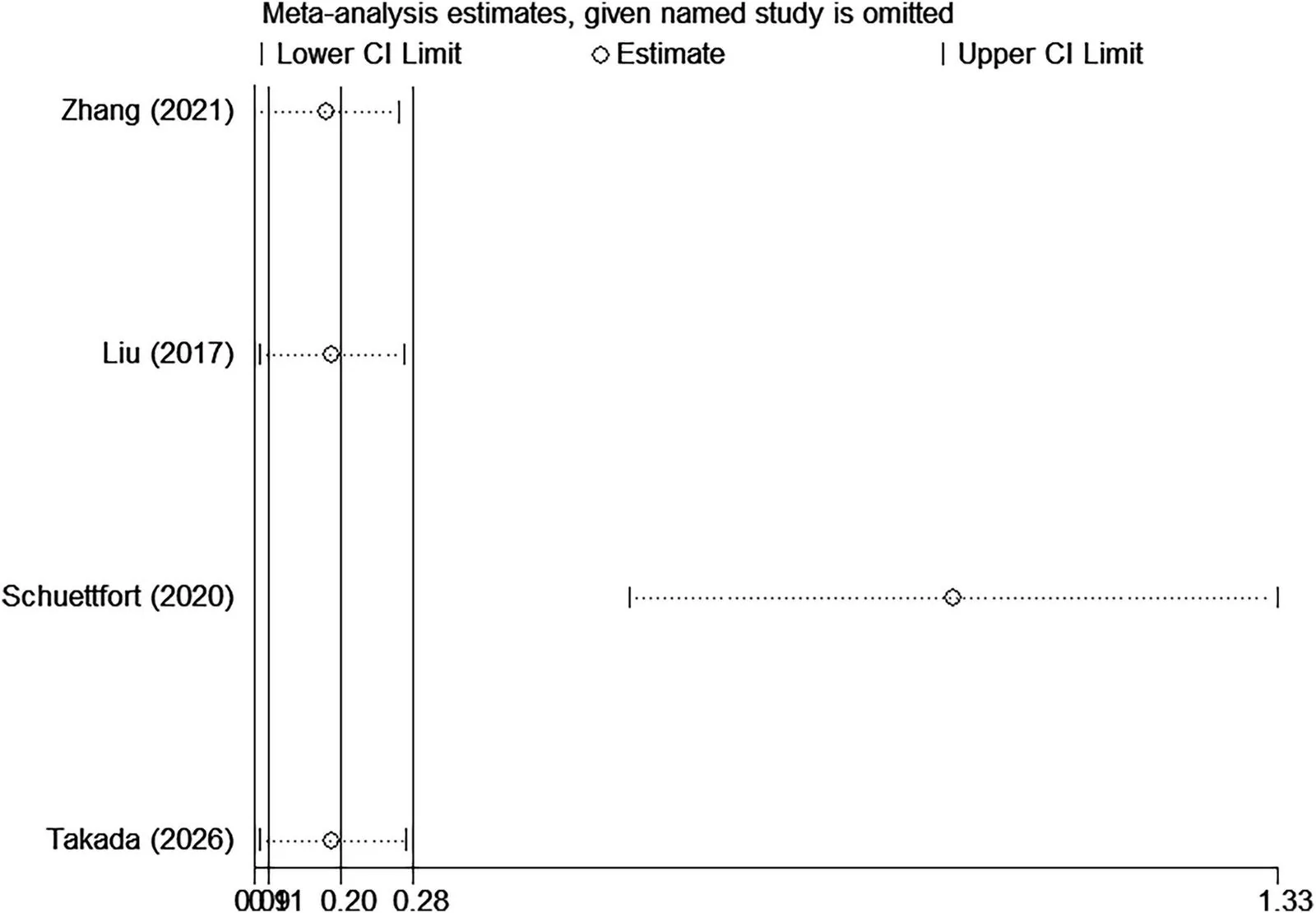
Sensitivity analysis for OS. OS, overall survival.

Sensitivity analysis was conducted by sequentially excluding individual studies on OS. The pooled hazard ratios did not change substantially after the omission of any single study, suggesting that the overall results were stable and reliable (Figure 3).

## Discussion

In the present meta-analysis, we systematically evaluated the association between the preoperative AGR and prognosis for bladder cancer. The pooled evidence indicates that a decreased preoperative AGR is associated with poor oncological outcomes, suggesting that the AGR may serve as a practical and clinically meaningful prognostic biomarker in this population. From a clinical perspective, the AGR has several advantages that make it attractive for routine use. First, the AGR is derived from standard preoperative biochemical tests and is inexpensive, noninvasive, and widely available. Second, the AGR can be assessed before definitive treatment, allowing early risk stratification and potentially informing perioperative optimization strategies. Third, the AGR may complement established clinicopathological factors rather than replace them. For example, prior studies of patients with bladder urothelial carcinoma treated with radical cystectomy revealed that the preoperative AGR was independently associated with long-term outcomes (12, 18), whereas a large NMIBC cohort reported that low AGR independently predicted progression to muscle-invasive disease, although its incremental discrimination beyond standard clinicopathologic features was limited (19).

The biological plausibility of the AGR as a prognostic marker lies in the fact that it simultaneously reflects two major host-related dimensions that are strongly linked to cancer outcomes: nutritional status and systemic inflammation. Systemic inflammation is increasingly recognized as a key contributor to tumor progression, metastasis, and treatment resistance (23). Mechanistically, tumor-promoting inflammation can support proliferation, angiogenesis, stromal remodeling, immune evasion, and metastatic colonization through cytokine- and chemokine-mediated pathways (24). Albumin is not only a marker of nutritional reserve but also reflects the host’s inflammatory and metabolic status. Reduced albumin levels may indicate malnutrition, increased inflammatory burden, impaired immune function, and decreased physiological reserve, all of which may weaken the patient’s ability to tolerate surgery, intravesical treatment, chemotherapy, or immunotherapy (25, 26). In contrast, the globulin fraction contains immunoglobulins, acute-phase proteins, and other inflammation-related proteins. Elevated globulin levels may therefore reflect chronic immune activation, tumor-associated inflammation, and immune dysregulation. In bladder cancer, persistent tumor-promoting inflammation may facilitate tumor cell proliferation, angiogenesis, invasion, immune escape, and metastatic progression. Thus, a low AGR may represent a combined state of reduced nutritional reserve and enhanced inflammatory burden, providing a plausible biological explanation for its association with poor oncological outcomes.

Our findings are also consistent with the broader literature on the AGR in urothelial malignancies. A previous meta-analysis in urothelial carcinoma reported that a low pretreatment AGR was associated with poor OS, CSS, and RFS, particularly in patients with non-metastatic UC (27). In addition, evidence from patients with advanced UC treated with pembrolizumab suggests that the AGR may retain prognostic significance even in contemporary systemic therapy settings (28). Taken together, these data support the view that the AGR is not merely a surgery-specific marker but may reflect a more general host-tumor interaction relevant across disease stages and treatment modalities.

Several issues may explain the heterogeneity across the studies included in our meta-analysis. The most important source is likely inconsistency in AGR cutoff values, which can substantially affect risk classification and pooled effect size estimates (18, 21, 22). Additional contributors include differences in study populations (NMIBC vs. MIBC/high-grade disease), treatment strategies (TURBT/intravesical therapy vs. radical cystectomy vs. systemic therapy), endpoint definitions, follow-up duration, and covariate adjustment models (12–14, 18, 19). A major challenge for the clinical implementation of the AGR is the inconsistency of cutoff values across studies. In the included studies, the AGR thresholds varied considerably, which may have led to different risk classifications even among patients with similar biochemical profiles. Therefore, although this meta-analysis supports the prognostic significance of the preoperative AGR, the absence of a standardized cutoff limits its immediate application as a uniform clinical decision-making tool. At present, the AGR should be interpreted as an adjunctive risk indicator rather than an independent criterion for treatment selection. Future prospective studies should use standardized AGR assessments, prespecified cutoff values, and external validation cohorts to determine clinically applicable thresholds.

Compared with previous studies, the present meta-analysis has several distinguishing features and potential advantages. First, whereas earlier evidence mainly came from individual retrospective cohorts or broader analyses of urothelial carcinoma, our study focused specifically on patients with bladder cancer and evaluated the prognostic significance of the preoperative AGR in this defined population. Second, this meta-analysis incorporated updated evidence and included multiple survival endpoints, including OS, CSS, PFS, and RFS, thereby providing a more comprehensive assessment of the prognostic value of the AGR. Third, by focusing on preoperative the AGR, our findings may have practical clinical relevance because the AGR can be obtained from routine biochemical tests before definitive treatment. In addition, the AGR is inexpensive, noninvasive, widely available, and easily applicable in routine clinical practice. Thus, AGR may serve as a complementary biomarker for early risk stratification and perioperative decision-making in patients with bladder cancer.

This study has several limitations. First, all the studies were retrospective, which may introduce the same bias despite multivariable adjustment. Second, the lack of a standardized AGR cutoff limits comparability across studies and hinders immediate clinical implementation. Third, some analyses may be based on a limited number of studies for specific endpoints, which may have reduced the robustness of the subgroup analyses and publication bias assessments. Fourth, differences in reporting standards and adjustment variables across studies may have influenced the pooled estimates. These limitations underscore the need for cautious interpretation of the results.

In conclusion, our meta-analysis suggested that a low preoperative AGR is significantly associated with poor prognosis for bladder cancer. Nevertheless, further high-quality prospective studies are needed to confirm its clinical utility.

## Data Availability

The original contributions presented in this study are included in this article/supplementary material, further inquiries can be directed to the corresponding author.

## References

[1] Mariappan P Johnston A Padovani L Clark E Trail M Hamid S et al. Enhanced quality and effectiveness of transurethral resection of bladder tumour in non-muscle-invasive bladder cancer: a multicentre real-world experience from scotland’s quality performance indicators programme. *Eur Urol*. (2020) 78:520–30. 10.1016/j.eururo.2020.06.051 32690321

[2] Alfred Witjes J Lebret T Compérat EM Cowan NC De Santis M Bruins HM et al. Updated 2016 EAU guidelines on muscle-invasive and metastatic bladder cancer. *Eur Urol*. (2017) 71:462–75. 10.1016/j.eururo.2016.06.020 27375033

[3] Yu EY Wesselius A Mehrkanoon S Goosens M Brinkman M van den Brandt P et al. Vegetable intake and the risk of bladder cancer in the bladder cancer epidemiology and nutritional determinants (BLEND) international study. *BMC Med*. (2021) 19:56. 10.1186/s12916-021-01931-8 33685459 PMC7942172

[4] Nakamura Y Imada A Fukugaki A Kanto S Yamaura T Kinjo Y et al. Association of nutritional risk and systemic inflammation with survival in patients with colorectal cancer who underwent curative surgery. *Clin Nutr ESPEN*. (2022) 49:417–24. 10.1016/j.clnesp.2022.03.011 35623847

[5] Ohanoglu Cetinel K Guner G Karabudak CB Zeynalli E Yavuz Sen S. Dynamic changes in prognostic nutritional index and systemic immune-inflammation index predict survival following debulking surgery in ovarian cancer. *Front Oncol*. (2026) 15:1720075. 10.3389/fonc.2025.1720075 41607545 PMC12836053

[6] Balkwill FR Mantovani A. Cancer-related inflammation: common themes and therapeutic opportunities. *Semin Cancer Biol*. (2012) 22:33–40. 10.1016/j.semcancer.2011.12.005 22210179

[7] Hoft SG Brennan M Carrero JA Jackson NM Pretorius CA Bigley TM et al. Unveiling cancer-related metaplastic cells in both *helicobacter* pylori infection and autoimmune gastritis. *Gastroenterology*. (2025) 168:53–67. 10.1053/j.gastro.2024.08.032 39236896 PMC11663102

[8] Zhang B Yu W Zhou LQ He ZS Shen C He Q et al. Prognostic significance of preoperative albumin-globulin ratio in patients with upper tract urothelial carcinoma. *PLoS One*. (2015) 10:e0144961. 10.1371/journal.pone.0144961 26681341 PMC4682975

[9] Roberts WS Delladio W Price S Murawski A Nguyen H. The efficacy of albumin-globulin ratio to predict prognosis in cancer patients. *Int J Clin Oncol.* (2023) 28:1101–11. 10.1007/s10147-023-02380-4 37421476

[10] He K Xu F Xiang L Luo Y. Albumin-globulin ratio is a predictive biomarker of antitumour effect of immune checkpoint inhibitors in cancer patients. *Ann Med*. (2025) 57:2591219. 10.1080/07853890.2025.2591219 41283335 PMC12646090

[11] Chung JW Park DJ Chun SY Choi SH Lee JN Kim BS et al. The prognostic role of preoperative serum albumin/globulin ratio in patients with non-metastatic renal cell carcinoma undergoing partial or radical nephrectomy. *Sci Rep*. (2020) 10:11999. 10.1038/s41598-020-68975-3 32686760 PMC7371633

[12] Liu Z Huang H Li S Yu W Li W Jin J et al. The prognostic value of preoperative serum albumin-globulin ratio for high-grade bladder urothelial carcinoma treated with radical cystectomy: a propensity score-matched analysis. *J Cancer Res Ther*. (2017) 13:837–43. 10.4103/jcrt.JCRT_237_17 29237914

[13] Niwa N Matsumoto K Ide H Nagata H Oya M. Prognostic value of pretreatment albumin-to-globulin ratio in patients with non-muscle-invasive bladder cancer. *Clin Genitourin Cancer*. (2018) 16:e655–61. 10.1016/j.clgc.2017.12.013 29366633

[14] Oh JS Park DJ Byeon KH Ha YS Kim TH Yoo ES et al. Decrease of preoperative serum albumin-to-globulin ratio as a prognostic indicator after radical cystectomy in patients with urothelial bladder cancer. *Urol J*. (2021) 18:66–73. 10.22037/uj.v16i7.6350 33515214

[15] Page MJ McKenzie JE Bossuyt PM Boutron I Hoffmann TC Mulrow CD et al. Updating guidance for reporting systematic reviews: development of the PRISMA 2020 statement. *J Clin Epidemiol*. (2021) 134:103–12. 10.1016/j.jclinepi.2021.02.003 33577987

[16] Norris JM Simpson BS Ball R Freeman A Kirkham A Parry MA et al. A modified Newcastle-Ottawa scale for assessment of study quality in genetic urological research. *Eur Urol*. (2021) 79:325–6. 10.1016/j.eururo.2020.12.017 33375994

[17] Hamling J Lee P Weitkunat R Ambühl M. Facilitating meta-analyses by deriving relative effect and precision estimates for alternative comparisons from a set of estimates presented by exposure level or disease category. *Stat Med*. (2008) 27:954–70. 10.1002/sim.3013 17676579

[18] Liu J Dai Y Zhou F Long Z Li Y Liu B et al. The prognostic role of preoperative serum albumin/globulin ratio in patients with bladder urothelial carcinoma undergoing radical cystectomy. *Urol Oncol.* (2016) 34:e1–484. 10.1016/j.urolonc.2016.05.024 27341738

[19] Quhal F Pradere B Laukhtina E Sari Motlagh R Mostafaei H Mori K et al. Prognostic value of albumin to globulin ratio in non-muscle-invasive bladder cancer. *World J Urol*. (2021) 39:3345–52. 10.1007/s00345-020-03586-1 33496841 PMC8510920

[20] Schuettfort VM Andrea D Quhal F Mostafaei H Laukhtina E Mori K et al. Impact of preoperative serum albumin-globulin ratio on disease outcome after radical cystectomy for urothelial carcinoma of the bladder. *Urol Oncol.* (2021) 39:e5–235. 10.1016/j.urolonc.2020.11.005 33189530

[21] Zhang W Yang F Kadier A Chen Y Yu Y Zhang J et al. Development of nomograms related to inflammatory biomarkers to estimate the prognosis of bladder cancer after radical cystectomy. *Ann Transl Med*. (2021) 9:1440. 10.21037/atm-21-4097 34733992 PMC8506704

[22] Takada H Ikeda M Tanaka R Samejima N Harano T Kobayashi S et al. Prognostic effect of preoperative albumin-to-globulin ratio in patients with non-metastatic bladder cancer undergoing radical cystectomy. *Oncology*. (2026) 104:880–90. 10.1159/000550892 41649974

[23] Wang M Chen S He X Yuan Y Wei X. Targeting inflammation as cancer therapy. *J Hematol Oncol*. (2024) 17:13. 10.1186/s13045-024-01528-7 38520006 PMC10960486

[24] Xie Y Liu F Wu Y Zhu Y Jiang Y Wu Q et al. Inflammation in cancer: therapeutic opportunities from new insights. *Mol Cancer*. (2025) 24:51. 10.1186/s12943-025-02243-8 39994787 PMC11849313

[25] Cabrerizo S Cuadras D Gomez-Busto F Artaza-Artabe I Marín-Ciancas F Malafarina V. Serum albumin and health in older people: review and meta analysis. *Maturitas*. (2015) 81:17–27. 10.1016/j.maturitas.2015.02.009 25782627

[26] Truong A Hanna MH Moghadamyeghaneh Z Stamos MJ. Implications of preoperative hypoalbuminemia in colorectal surgery. *World J Gastrointest Surg*. (2016) 8:353–62. 10.4240/wjgs.v8.i5.353 27231513 PMC4872063

[27] Xia Z Fu X Li J Wu J Niu C Xu Y et al. Prognostic value of pretreatment serum albumin-globulin ratio in urothelial carcinoma: A systematic review and meta-analysis. *Front Oncol.* (2022) 12:992118. 10.3389/fonc.2022.992118 36052239 PMC9424645

[28] Takada H Ikeda M Tanaka R Samejima N Harano T Kobayashi S et al. Prognostic effect of preoperative albumin-to-globulin ratio in patients with non-metastatic bladder cancer undergoing radical cystectomy. *Oncology*. (2026) 8:880–90. 10.1159/000550892 41649974

